# Post‐endoscopic retrograde cholangiopancreatography pancreatitis assessed using criteria for acute pancreatitis

**DOI:** 10.1002/jgh3.12687

**Published:** 2021-12-06

**Authors:** Azumi Suzuki, Koji Uno, Kojiro Nakase, Koichiro Mandai, Bunji Endoh, Koki Chikugo, Takumi Kawakami, Takahiro Suzuki, Yoshitaka Nakai, Kiyonori Kusumoto, Yoshio Itokawa, Osamu Inatomi, Shigeki Bamba, Yoshinori Mizumoto, Kiyohito Tanaka

**Affiliations:** ^1^ Department of Gastroenterology Kyoto Second Red Cross Hospital Kyoto Japan; ^2^ Department of Gastroenterology National Hospital Organization Kyoto Medical Center Kyoto Japan; ^3^ Department of Gastroenterology Japanese Red Cross Kyoto Daiichi Hospital Kyoto Japan; ^4^ Digestive Disease Center, Department of Gastroenterology and Hepatology Kyoto Katsura Hospital Kyoto Japan; ^5^ Division of Gastroenterology, Department of Medicine Shiga University of Medical Science Otsu Japan; ^6^ Present address: Department of Gastroenterology Hamamatsu Medical Center Shizuoka Japan; ^7^ Present address: Department of Gastroenterology Kyoto Okamoto Memorial Hospital Kyoto Japan; ^8^ Present address: Center for Gastroenterology Teine Keijinkai Hospital Sapporo Japan; ^9^ Present address: Department of Gastroenterology Municipal Tsuruga Hospital Fukui Japan; ^10^ Present address: Suzuki Naika Iin Kyoto Japan

**Keywords:** acute pancreatitis, cohort studies, endoscopic retrograde cholangiopancreatography, intraductal ultrasonography, post‐endoscopic retrograde cholangiopancreatography pancreatitis

## Abstract

**Background and Aim:**

International consensus on the definition and classification of post‐endoscopic retrograde cholangiopancreatography (ERCP) pancreatitis (PEP) has been reached. However, the diagnosis and severity of PEP are often assessed according to the diagnostic criteria and classification for acute pancreatitis (AP). This study determined the incidence, severity, and risk factors of PEP diagnosed according to the diagnostic criteria and classification for AP in a large cohort.

**Methods:**

This prospective, multicenter, observational cohort study conducted at five high‐volume centers included 1932 patients who underwent ERCP‐related procedures. The incidence, severity, and risk factors for PEP were evaluated.

**Results:**

PEP occurred in 142 patients (7.3%); it was mild in 117 patients (6.0%) and severe in 25 patients (1.3%). According to the Cotton criteria, PEP occurred in 87 patients (4.5%); it was mild in 54 patients (2.8%), moderate in 20 patients (1.0%), and severe in 13 patients (0.7%). In the multivariate analysis, female sex (odds ratio [OR] 2.239; 95% confidence interval [CI] 1.546–3.243), naïve papilla (OR 3.047; 95% CI 1.803–5.150), surgically‐altered gastrointestinal anatomy (OR 2.538; 95% CI 1.342–4.802), procedure time after reaching the papilla (OR 1.009; 95% CI 1.001–1.017), pancreatic duct injection (OR 2.396; 95% CI 1.565–3.669), and intraductal ultrasonography (OR 1.641; 95% CI 1.024–2.629) were independent risk factors.

**Conclusion:**

According to the diagnostic criteria and classification for AP, the incidence of PEP was higher than that according to the Cotton criteria and the severity of PEP tended to be severe.

## Graphical abstract

According to the diagnostic criteria for acute pancreatitis (AP), the incidence of post‐endoscopic retrograde cholangiopancreatography pancreatitis (PEP) was higher than that with the Cotton criteria; the incidence rate of PEP was equivalent to that in previous reports. More patients were diagnosed with severe PEP using the Japanese severity criteria for AP. The risk factors of PEP were female sex, naïve papilla, surgically altered gastrointestinal anatomy, procedure time after reaching the papilla, pancreatic duct injection, and intraductal ultrasonography.

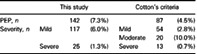

## Introduction

Procedures related to endoscopic retrograde cholangiopancreatography (ERCP) play a major role in the diagnosis and treatment of pancreatobiliary diseases. Post‐ERCP pancreatitis (PEP) remains the most common and severe complication of ERCP. The condition of PEP ranges from mild, requiring only intravenous fluid therapy, to severe, requiring intensive care. Even if PEP is initially diagnosed as mild, it may become severe during the clinical course. It is important to accurately diagnose and evaluate the severity of PEP, promptly start appropriate treatment, and repeatedly evaluate its severity during the course. Previous studies have reported that the incidence of PEP is 3.5–9.7%.^1^, ^2^ At present, there are no established diagnostic criteria for PEP, and most previous studies used the diagnostic and classification criteria proposed by Cotton *et al*.^3^: (i) new or worsened abdominal pain; (ii) serum amylase at least three times the upper limit of normal, measured more than 24 h after the procedure; and (iii) new or prolongation of hospitalization for at least 2 days. The Cotton criteria have obtained international consensus and persistent upper abdominal pain and elevated serum amylase are important findings of PEP.

Nevertheless, it has been reported that when lipase levels and image findings that are usually used for the diagnosis of acute pancreatitis (AP) are added to the criteria, the number of diagnosed PEP cases increased, and 41.9% of the PEP cases were overlooked with the consensus criteria alone.^4^ Another study reported that 37% of patients who had hyperamylasemia without abdominal pain after ERCP was diagnosed with pancreatitis based on computed tomography (CT).^5^ In addition, regarding severity assessment, the Cotton criteria have another problem in that they do not accurately evaluate severity in the early phase or allow repeated assessment in a short period. Therefore, the severity assessment for AP proposed by the Japanese Ministry of Health, Labour, and Welfare (Japanese severity criteria for AP) (Table 1),^6^ which allows the evaluation of the severity of pancreatitis in the early phase and repeated assessment, is usually used for severity assessment of PEP in Japan. Although the diagnostic criteria and severity classification of AP are often used to assess PEP in clinical practice, few studies^7^, ^8^ have used these clinical criteria in the epidemiological study of PEP.

**Table 1:** Japanese severity scoring system for acute pancreatitis of the Ministry of Health, Labour, and Welfare of Japan (2008 revision)

Prognostic factors (one point for each factor)	CT grade based on contrast‐enhanced CT
Base excess ≦−3 mEq/L or shock (systolic blood pressure <80 mmHg) PaO_2_ ≦ 60 mmHg (room air) or respiratory failure (respiratory assistance needed) BUN ≧ 40 mg/dL or (or creatinine ≧ 2.0 mg/dL) or oliguria (daily urine output <400 mL even after intravenous fluid resuscitation) LDH ≧ twice upper limit of normal Platelet count ≦ 100 000/mm^3^ Serum Ca ≦ 7.5 mg/dL CRP ≧ 15 mg/dL Number of positive measures in SIRS criteria ≧3 Age ≧ 70 years	Extrapancreatic progression of inflammation Anterior pararenal space, 0 point Root of mesocolon, 1 point Beyond lower pole of kidney, 2 points 2. Hypoenhanced lesion of the pancreas The pancreas is conveniently divided into three segments (head, body, and tail) Localized in each segment or only surrounding the pancreas, 0 point Extends to 2 segments, 1 point Occupies 2 whole segments or more, 2 points 1 + 2 = total score Total score = 0 or 1, Grade 1 Total score = 2, Grade 2 Total score = 3 or more, Grade 3
Assessment of severity If prognostic factor score is ≧3, or CT grade is ≧2, the acute pancreatitis is evaluated as ‘severe’	

Measures in SIRS criteria include body temperature >38 or <36°C, heart rate >90 beats/min, respiratory rate >20 breaths/min or PaCO_2_ <32 torr, and white blood cell counts >12 000 cells/mm^3^, <4000 cells/mm^3^, or >10% immature (band) forms.

BUN, blood urea nitrogen; CRP, C‐reactive protein; CT, computed tomography; LDH; lactate dehydrogenase, SIRS, systemic inflammatory response syndrome.

Therefore, in this study, we considered PEP as AP that occurred after ERCP. We conducted this prospective multicenter study to determine the incidence, severity, and risk factors of PEP that is diagnosed according to the diagnostic criteria and classification for AP in a large cohort.

## Methods

This is a prospective multicenter observational cohort study conducted at five high‐volume centers in Kyoto and Shiga prefecture of western Japan. The incidence and severity of PEP that had been diagnosed according to diagnostic and severity criteria for AP were investigated and compared to those, which were obtained according to the consensus criteria proposed by Cotton *et al*. Risk factors for PEP were statistically analyzed using multivariate analysis. The variables investigated in this study are shown in Table 2. The protocol of this study was approved by the ethics committees of all participating centers and registered in the University hospital Medical Information Network (UMIN) clinical trial registration system (UMIN000024813). This study was conducted in accordance with the ethical guidelines of the Declaration of Helsinki.

**Table 2:** Investigated risk factors of PEP

Patient‐related factors		
Female sex	Previous acute pancreatitis	Naïve papilla
Younger age (<50 years of age)	Normal serum bilirubin	Peripapillary diverticulum
ASA grade	Acute cholangitis	Surgically altered gastrointestinal anatomy
Procedure‐related factors		
Therapeutic ERCP	Minor papilla cannulation	Precut sphincterotomy
Emergency ERCP	Pancreatic duct injection	Endoscopic stone extraction
Procedure time after reaching papilla	Pancreatic guidewire passage	Endoscopic biliary drainage
ERCP by trainee	Intraductal ultrasonography	Pancreatic duct brushing cytology
Contrast‐guided cannulation	Endoscopic sphincterotomy	Pancreatic stenting
Cannulation attempts (≧10)	Endoscopic papillary balloon dilatation	Prophylactic pancreatic stent placement

ASA; American Society of Anesthesiologists; ERCP; endoscopic retrograde cholangiopancreatography; PEP, post‐endoscopic retrograde cholangiopancreatography pancreatitis.

### Patients

All patients who underwent ERCP‐related procedures at the centers between February 2015 and May 2016 were enrolled in a registry and the data on patient characteristics, indications for ERCP, findings of procedures, and adverse events were collected prospectively. We excluded patients who had AP at the time of ERCP, who had undergone biliary reconstruction, or in whom the endoscopist failed to reach the major papilla during ERCP. ERCP‐related procedures were performed in accordance with the strategies of each center, and blood tests were performed 2 h after ERCP and 18 h (the following morning) in all patients. Written informed consent was obtained from all the patients prior to registration.

### Definitions

PEP was defined as the presence of at least two of the following three manifestations based on diagnostic criteria for AP^9^, ^10^: (i) elevated levels of serum amylase; (ii) abdominal pain lasting more than 24 h; and (iii) characteristics findings of AP on CT. The elevation of serum amylase levels was considered significant when it was elevated to more than three times the upper limit of normal according to the consensus criteria.^3^ CT findings that confirm the diagnosis of AP include focal or diffuse enlargement of the pancreas, heterogeneity of pancreatic parenchyma, peripancreatic stranding, pancreatic or peripancreatic fluid collections, pancreatic necrosis, and peripancreatic fat necrosis. Two expert radiological diagnosticians who were blinded to clinical information independently assessed CT images and confirmed the presence or absence of AP.^11^, ^12^, ^13^, ^14^ The severity of PEP was assessed according to the Japanese severity assessment for AP.^6^

### Statistical analysis

A multivariable logistic regression analysis was used to identify the independent risk factors for PEP. First, Variables in Table 2 were assessed using univariate analysis with the χ^2^ test for categorical variables and Mann–Whitney *U* test for continuous variables. The variables entered into the logistic regression model were chosen referring to prior reports and results of the preceding univariable analyses and considering the scientific plausibility and the clinical meaningfulness of the association. The number of variables entered into the logistic regression model was determined according to the rule of thumb that a logistic model should be used with a minimum of 10 events per predictor variable. *P*‐values <0.05 were considered significant. Statistical analyses were performed using IBM SPSS statistics 22 (IBM Corp, Armonk, NY, USA).

## Results

**Figure 1:**
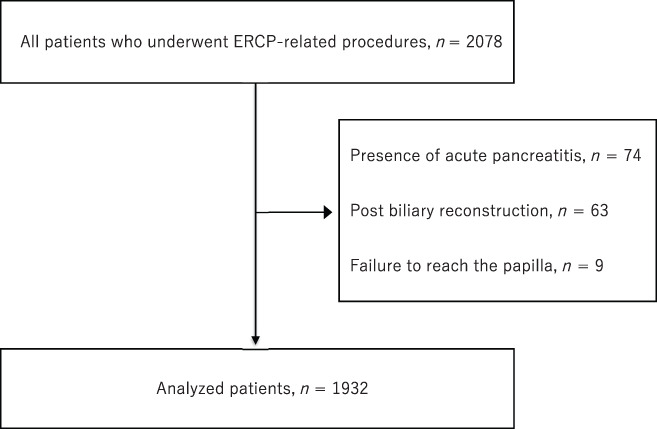
Flowchart of the registered patients. There were 2078 patients who received endoscopic retrograde cholangiopancreatography (ERCP)‐related procedures. We excluded 74 patients who already had acute pancreatitis, 63 who had undergone biliary reconstruction, and 9 in whom the endoscope could not reach the papilla of Vater. We analyzed 1932 patients.

Overall, 2078 patients were enrolled in the registry. We excluded 74 patients who already had AP, 63 who had undergone biliary reconstruction, and 9 in whom the endoscope could not reach the papilla of Vater; 1932 patients were finally analyzed (Fig. 1).

Table 3 displays the patient characteristics. The mean age of the patients was 72.9 years, and 774 (40.1%) were female; 87.4% of the patients had American Society of Anesthesiologists (ASA) grade I or II, 13.5% had a history of AP, 34.7% had obstructive jaundice, 54.6% had naïve papillae, and 5.5% had a surgically‐altered gastrointestinal (GI) anatomy. The most common indication for ERCP was choledocholithiasis, accounting for 46.0% of the total. The proportion of patients with malignant diseases was 36.5%.

**Table 3:** Patient characteristics

	*n* (%)	
Mean age (years old)	72.9	
Female	774	(40.1)
ASA grade		
1	924	(47.8)
2	766	(39.6)
3	227	(11.7)
4	15	(0.8)
Previous acute pancreatitis	261	(13.5)
Obstructive jaundice	671	(34.7)
Acute cholangitis	591	(30.6)
Naïve papilla	1054	(54.6)
Peripapillary diverticulum	498	(25.8)
Surgically altered gastrointestinal anatomy	107	(5.5)
Billroth‐I	44	(2.3)
Billroth‐II	22	(1.1)
Roux‐en‐Y	41	(2.1)
Indication of ERCP		
Choledocholithiasis	888	(46.0)
Chronic pancreatitis/pancreatic stone	131	(6.8)
Benign biliary stenosis	66	(3.4)
Acute cholangitis	50	(2.6)
Pancreatic cancer	261	(13.5)
Bile duct cancer	248	(12.8)
Gallbladder cancer	36	(1.9)
Other malignant tumor	63	(3.3)
Intraductal papillary mucinous neoplasm	49	(2.5)
Papillary tumor	29	(1.5)
Others	111	(5.7)

ASA, American Society of Anesthesiologists; ERCP, endoscopic retrograde cholangiopancreatography.

Table 4 displays the characteristics of ERCP‐related procedures. Therapeutic ERCP accounted for large proportion of patients at 89.1%. The proportion of patients undergoing emergency ERCP was 22.7% and that of those undergoing ERCP by trainees with less than 5 years of ERCP experience was 60.4%. The average procedure time was 37.9 min.

**Table 4:** Characteristics of procedures

	*n* (%)	
Therapeutic ERCP	1722	(89.1)
Emergency ERCP	438	(22.7)
ERCP by trainees	1166	(60.4)
Mean procedure time (min)	37.9	
Cannulation method		
Wire‐guided	1041	(53.9)
Contrast‐guided	869	(45.0)
Others	22	(1.1)
Cannulation attempts		
1–3	1243	(64.3)
4–9	376	(19.5)
≧10	313	(16.2)
Minor papilla cannulation	19	(1.0)
Pancreatic duct injection	595	(30.8)
Pancreatic guidewire passage	469	(24.3)
Intraductal ultrasonography	225	(11.6)
Endoscopic sphincterotomy	484	(25.1)
Endoscopic papillary balloon dilatation	136	(7.0)
Precut sphincterotomy	31	(1.6)
Endoscopic stone extraction	587	(30.4)
Endoscopic biliary drainage	1135	(58.7)
Pancreatic duct brushing cytology	30	(1.6)
Pancreatic stenting	234	(12.1)
Prophylactic pancreatic stenting	99	(5.1)

ERCP, endoscopic retrograde cholangiopancreatography.

Wire‐guided cannulation was the first choice for cannulation at three of the five centers and contrast‐guided cannulation was the first choice at two centers. Wire‐guided cannulation was performed in 1041 patients (53.9%) and contrast‐guided cannulation was used in 869 (45.0%). In approximately two‐thirds of the patients, cannulation was successful within 1–3 attempts. Including unintentional contrast material injection and guidewire insertion into the pancreatic duct, pancreatic duct injection was performed in 595 patients (30.8%) and pancreatic guidewire passage was performed in 469 patients (24.3%). Intraductal ultrasonography (IDUS) was performed in 225 patients (11.6%). The morning after ERCP, serum amylase levels were elevated to more than the upper limit of normal in 576 patients (29.8%) and to more than three times the upper limit of normal in 207 patients (10.7%). Abdominal CT scan was performed in 444 of 576 patients (77.1%) with high amylase levels.

PEP occurred in 142 of 1932 patients (7.3%). The severity was mild in 117 patients (6.0%) and severe in 25 patients (1.3%) according to the Japanese severity criteria for AP. Using the Cotton consensus criteria, PEP was diagnosed in 87 patients (4.5%), and the severity was mild in 54 patients (2.8%), moderate in 20 patients (1.0%), and severe in 13 patients (0.7%) (Table 5). Overall, among the 117 patients diagnosed with mild PEP according to the Japanese severity criteria for AP, 50 patients were diagnosed as having nonpancreatitis, 65 patients mild–moderate PEP, and two patients severe PEP according to the Cotton criteria. Among 25 patients diagnosed with severe PEP according to the Japanese severity criteria for AP, five patients were diagnosed with nonpancreatitis, nine patients mild–moderate PEP, and 11 patients severe PEP according to the Cotton criteria.

**Table 5:** Incidence rate of PEP

	This study			Cotton's criteria		
PEP, *n*		142	(7.3%)		87	(4.5%)
Severity, *n*	Mild	117	(6.0%)	Mild	54	(2.8%)
				Moderate	20	(10.0%)
	Severe	25	(1.3%)	Severe	13	(0.7%)

PEP, post‐endoscopic retrograde cholangiopancreatography pancreatitis.

Regarding risk factors of PEP, in univariate analysis, female sex, naïve papilla, surgically altered GI anatomy, no coexistence of acute cholangitis, diagnostic ERCP, elective ERCP, procedure time after reaching the papilla, number of cannulation attempts, precut sphincterotomy, IDUS, pancreatic duct injection, pancreatic guidewire passage, and prophylactic pancreatic stenting were significant risk factors (Table 6).

**Table 6:** Risk factors of post‐ERCP pancreatitis

	Univariate analysis^†^			Multivariate analysis^‡^		
	OR	95% CI	*P* value	OR	95% CI	*P* value
Patient‐related risk factors						
Female sex	2.237	1.583–3.161	<0.001	2.239	1.546–3.243	<0.001
Younger age (<50 years of age)	0.732	0.292–1.834	0.504	0.703	0.271–1.822	0.468
ASA grade III or IV	0.879	0.516–1.497	0.638	—	—	—
Previous acute pancreatitis	0.626	0.349–1.125	0.114	0.881	0.462–1.683	0.702
Normal serum bilirubin	0.915	0.642–1.303	0.623	1.032	0.689–1.546	0.878
No coexistence of acute cholangitis	1.630	1.084–2.451	0.019	—	—	—
Naïve papilla	5.286	3.296–8.480	<0.001	3.047	1.803–5.150	<0.001
Surgically altered gastrointestinal anatomy	2.371	1.352–4.156	0.002	2.538	1.342–4.802	0.004
Peripapillary diverticulum	0.908	0.608–1.356	0.637	—	—	—
Procedure‐related risk factors						
Diagnostic ERCP	2.170	1.396–3.372	<0.001	—	—	—
Elective ERCP	1.750	1.087–2.817	0.020	1.364	0.801–2.324	0.254
Procedure time after reaching the papilla			<0.001^§^	1.009	1.001–1.017	0.035
ERCP by trainee	0.978	0.690–1.386	0.901	—	—	—
Contrast‐guided cannulation	1.273	0.903–1.794	0.168	—	—	—
Cannulation attempts (≧10)	3.155	2.183–4.561	<0.001	1.118	0.700–1.786	0.641
Minor papilla cannulation	1.490	0.341–6.514	0.646	—	—	—
Pancreatic duct injection	3.992	2.805–5.681	<0.001	2.396	1.565–3.669	<0.001
Pancreatic guidewire passage	2.989	2.111–4.233	<0.001	1.340	0.854–2.102	0.202
Intraductal ultrasonography	2.191	1.426–3.366	<0.001	1.641	1.024–2.629	0.040
Endoscopic sphincterotomy	1.189	0.812–1.740	0.373	—	—	—
Endoscopic papillary balloon dilatation	0.563	0.244–1.301	0.173	—	—	—
Precut sphincterotomy	3.815	1.615–9.016	0.006	1.028	0.389–2.717	0.956
Endoscopic stone extraction	0.704	0.473–1.049	0.083	—	—	—
Endoscopic biliary drainage	0.820	0.581–1.155	0.255	—	—	—
Pancreatic duct brushing cytology	2.577	0.971–6.837	0.064	—	—	—
Pancreatic stenting	1.285	0.791–2.087	0.310	—	—	—
Prophylactic pancreatic stenting	2.399	1.345–4.277	0.002	0.935	0.478–1.827	0.843

^†^ χ^2^ test.

^‡^ Logistic regression analysis.

^§^ Mann–Whitney *U* test.

ASA, American Society of Anesthesiologists; CI; confidence interval; ERCP, endoscopic retrograde cholangiopancreatography; OR, odds ratio.

Referring to this result and prior reports, 14 variables were entered into a logistic regression model to assess risk factor for PEP: female sex, younger age, previous AP, normal serum bilirubin, naïve papilla, surgically altered GI anatomy, elective ERCP, procedure time, number of cannulation attempts, precut sphincterotomy, IDUS, pancreatic duct injection, pancreatic guidewire passage, and prophylactic pancreatic stenting. In multivariate analysis, female sex (odds ratio [OR] 2.239; 95% confidence interval [CI] 1.546–3.243), naïve papilla (OR 3.047; 95% CI 1.803–5.150), surgically‐altered GI anatomy (OR 2.538; 95% CI 1.342–4.802), procedure time after reaching the papilla (OR 1.009; 95% CI 1.001–1.017), pancreatic duct injection (OR 2.396; 95% CI 1.565–3.669), and IDUS (OR 1.641; 95% CI 1.024–2.629) were independent risk factors (Table 6).

## Discussion

According to the diagnostic criteria and classification for AP, the incidence rate of PEP was 7.3% and that of severe PEP was 0.7%. Female sex, naïve papilla, surgically‐altered GI anatomy, procedure time after reaching the papilla, pancreatic duct injection, and IDUS were found to be the risk factors in multivariate analysis.

We diagnosed PEP based on diagnostic criteria for AP.^9^, ^10^ PEP was diagnosed in 142 patients (7.3%), while 87 patients (4.5%) were diagnosed as having PEP according to the Cotton consensus criteria. These incidence rates of PEP were not significantly different from that of previous reports (3.5–9.7%).^1^, ^2^ A total of 38.7% of patients with PEP were not diagnosed with PEP according to the consensus criteria, which was similar to the results presented by Artifon *et al*.^4^ Of the 55 patients who were not diagnosed with PEP based on the consensus criteria alone, 45 patients did not have persistent abdominal pain and 10 patients did not have significantly elevated serum amylase levels. Adding CT findings to the diagnostic criteria, PEP with poor clinical symptoms can be identified. This may lead to early detection of PEP and appropriate initial management to improve clinical course, but further studies are needed to confirm that.

We assessed the severity of PEP using Japanese severity criteria for AP. These criteria have been reported to correlate with in‐hospital mortality and to be useful for severity assessment of AP at the early stage of hospital admission.^15^ The severity of PEP was mild in 6.0% and severe in 1.3%, and severe PEP was occurred in 0.7% (13 patients) according to the Cotton criteria in this study. The incidence of severe PEP has been reported to be 0.3–0.5%.^1^, ^2^ The Japanese severity criteria for AP potentially deemed more cases as severe in contrast to the Cotton criteria. Another possibility is that PEP was diagnosed as severe in the early phase and appropriate initial treatment improved the clinical course. The revised Atlanta Classification^9^ has been also proposed as criteria for assessing the severity of AP. However, the revised classification that includes persistent organ failure as the key determinant of severity also poses difficulties for evaluating the severity in the early phase and repeated assessment as with the Cotton criteria. Because the pathophysiology of PEP may differ from that of AP, unique severity criteria need to be established for the pathophysiology of PEP.

We also assessed risk factors of PEP in this study. “No coexistence of acute cholangitis” and “diagnostic ERCP” were significant risk factors in the univariate analysis but these factors were not entered into a logistic regression analysis, because we considered that these associations are due to the association of these findings with procedure time and naive papilla, respectively, and are scientifically implausible.

In the multivariable analysis of this study, female sex, naïve papilla, surgically altered GI anatomy, procedure time after reaching the papilla, pancreatic duct injection, and IDUS were found to be independent risk factors. Female sex and pancreatic duct injection were considered risk factors for PEP in the American Society for Gastrointestinal Endoscopy guidelines^16^ and European Society Gastrointestinal Endoscopy guidelines^17^ among other reports.^18^, ^19^, ^20^ IDUS has been reported to be an independent risk factor for PEP in a retrospective study.^21^ In the report, Meister *et al*. suggested that papilledema of Vater caused by physical stimulation with an IDUS probe induces PEP. In our study, a naïve papilla was defined as a major papilla that has not undergone a prior endoscopic procedure such as endoscopic sphincterotomy (EST) or endoscopic papillary balloon dilation (EPBD) at the time of ERCP. Deep cannulation in patients with a naïve papilla is usually more difficult than in patients who have already undergone EST or EPBD and physical stimulation of a naive papilla is often stronger. Moreover, in many cases of naïve papillae, because the bile duct orifice and pancreatic duct orifice are not separated as in a post‐EST case, physical stimulation of the papilla can easily influence the pancreatic orifice. This may explain why the incidence of PEP increased in patients with a naïve papilla. A conventional forward‐viewing endoscope or a balloon enteroscope is usually used for ERCP in patients with Billroth II reconstruction or Roux‐en‐Y reconstruction. In such cases, when reaching the papilla of Vater, the papilla is often on the left or upper side of the screen and the view is upside down as compared to an ordinary ERCP view.^22^ Thus, physical stimulation of the papilla is likely to be more extensive because of the difficult positioning of the papilla and the lack of the elevator that facilitates delicate maneuvers of devices. Furthermore, a duodenoscope is used in patients with Billroth I reconstruction; however, deformation of the duodenum and common bile duct often makes cannulation difficult and increases the risk of developing PEP. Moreover, physical stimulation of the papilla of Vater can occur not only during cannulation but also during the entire procedure. This suggests that longer procedure times after reaching the papilla correlate with stronger irritation to the papilla. It has been reported that procedure time of 30 min or more is a risk factor of PEP.^23^ Previous pancreatitis, younger age, normal serum bilirubin, and pancreatic guidewire passage have been reported to be independent risk factors for PEP^16^, ^17^; however, in the present study, we did not identify these as significant risk factors for PEP.

This study had some limitations that need to be taken into account while interpreting the results. This study is limited by its multicenter prospective observational design, and there was no standardized protocol for ERCP‐related procedures. Therefore, incidence of PEP and a correlation between each risk factor and PEP might be confounded by unmeasured factors. For example, we did not investigate the amount of hydration in our study; however, it has been suggested that aggressive hydration with lactated Ringer's solution reduces the incidence of PEP.^24^, ^25^, ^26^, ^27^ The amount of hydration possibly confounded the development of PEP. In addition, rectal administration of nonsteroidal anti‐inflammatory drugs (NSAIDs) and prophylactic pancreatic stent placement, both of which have been shown to be effective in preventing PEP,^20^, ^28^, ^29^, ^30^, ^31^, ^32^ was performed only in high‐risk patients according to the judgment of the operator. As a result, the number of patients who received rectal NSAIDs was small and rectal NSAIDs were excluded from the variables investigated. Prophylactic pancreatic stenting has been recognized as a risk factor for PEP in univariate analysis. Finally, selection bias may have influenced the results. However, it was confirmed that the amount of hydration before and after ERCP and the selection of high‐risk cases for PEP did not differ significantly among the five institutions at a pre‐study meeting, and thus, the effects of confounding are likely to be small. In order to evaluate the preventive effects of these factors, it is necessary to investigate PEP using a protocol for prophylactic treatment in patients other than those at high risk and diagnose PEP based on the criteria for AP.

In conclusion, according to the diagnostic criteria for AP, the incidence of PEP was higher than that with the Cotton criteria; the incidence rate of PEP was equivalent to that in previous reports. More patients were diagnosed with severe PEP using the Japanese severity criteria for AP. The risk factors of PEP were female sex, naïve papilla, surgically altered GI anatomy, procedure time after reaching the papilla, pancreatic duct injection, and IDUS.
